# A cross-sectional survey on exploring adherence to diabetes treatment regimens: Insights from Hail region, Saudi Arabia

**DOI:** 10.1097/MD.0000000000049485

**Published:** 2026-08-14

**Authors:** Mukhtar Ansari

**Affiliations:** a Department of Clinical Pharmacy, College of Pharmacy, University of Ha’il, Hail, Saudi Arabia.

**Keywords:** diabetes mellitus, hail region, medication adherence, non-adherence, Saudi Arabia

## Abstract

Diabetes mellitus is a rapidly growing public health concern globally, and medication adherence plays an important role in managing diabetes. This study was carried out with the purpose of assessing patient adherence to anti-diabetic medication among the people of the Hail region, which is associated with long-term complications due to diabetes. This cross-sectional study was conducted from May to June 2023 using an online questionnaire among 254 diabetes patients in the Hail region of Saudi Arabia. The questionnaire assessed socio-demographic factors and medication adherence, which was scored and categorized into low, intermediate, and high adherence. Data were analyzed using Mann–Whitney *U* and Kruskal–Wallis tests to identify associations. Over 84% of participants showed low to intermediate adherence, whereas only 15.8% were highly adherent. Treatment inconvenience, forgetfulness, and discontinuation due to side effects were among the main barriers to medication adherence. There was a strong correlation (*P* < .001) between improved medication adherence and age, education, and marital status. However, gender and body weight did not show any significant correlations. Diabetes patients in Hail have suboptimal medication adherence, which is impacted by behavioral and demographic factors. It is essential to address barriers through patient education, regimen simplification, and culturally tailored strategies to improve adherence and avoid long-term complications.

## 1. Introduction

Diabetes mellitus has emerged as a significant global public health issue, with its increasing prevalence. According to the 11th Edition of the International Diabetes Federation Atlas 2025, there were 588.7 million people globally living with diabetes in 2024. By 2050, this number is expected to increase by 45% to around 852.5 million. The increase is even more pronounced in the Middle East and North Africa region, where it is 92% – more than double the global increase – and the second highest after Africa, which is expected to see a 142% rise. The Middle East and North Africa region had about 84.7 million individuals with diabetes in 2024, anticipated to grow to 162.6 million by 2050.^[1]^ With 5.3 million people living with diabetes between the ages of 20 and 79 in 2024, Saudi Arabia is one of the top 5 countries in the world. Additionally, 43.6% of individuals in this age group remain undiagnosed. This number is projected to increase to 9.5 million by 2050,^[1]^ and the leading causes behind this are rapid urbanization, more sedentary lifestyles, and changes in dietary patterns.^[2–4]^ With a prevalence rate of 10.8%, the Hail region has the second-highest rate of diabetes after Makkah (11%). The increasing incidence of diabetes in this region necessitates the importance of effective management approaches to prevent diabetes-related complications.^[5]^

Maintaining ideal blood glucose levels is still challenging, even with the availability of numerous anti-diabetic drugs and well-established treatment plans. Glucagon-like peptide-1 receptor agonists are among the newer antidiabetic medications gaining attention in the management of diabetes. They bear numerous advantages over conventional antidiabetic medications in terms of better pharmacokinetic profiles and efficacy. Apart from their primary role in managing diabetes, they play a beneficial role in weight reduction and reduce cardiovascular and related complications. They have also been found to have a beneficial role in renal, respiratory tract, and even cognitive function; however, they are associated with increased risks of adverse effects.^[6,7]^ However, adverse effects, along with the higher cost of glucagon-like peptide-1 receptor agonists, could be reasons for the discontinuation of therapy. Thus, there is a need for advanced clinical research.^[8,9]^ The degree to which patients follow their recommended medication regimens is one of the major determinants affecting treatment success.^[10,11]^ Inadequate glycemic control, a higher risk of complications (such as heart attacks and strokes, nephropathy, retinopathy, and kidney failure), more hospital admissions, and higher healthcare costs can all result from poor medication adherence.^[12,13]^ Non-adherence is caused by a number of factors, including drug side effects, patient ignorance, cultural influences, and socioeconomic factors,^[14–16]^ highlighting the complexity of this issue within the region.

The urgent necessity to investigate the trends and underlying causes of medication adherence among diabetes patients in the Hail region is the driving force for this study. Understanding these factors can help healthcare providers and policymakers in developing focused plans to improve compliance, which will mitigate the overall burden of disease and improve patients’ quality of life. In order to reduce long-term problems from diabetes, this study aimed to evaluate patient adherence to anti-diabetic medication among the people of the Hail region.

## 2. Materials and methods

### 2.1. Study design and setting

This cross-sectional study was carried out between May 2023 and June 2023 in Hail, Saudi Arabia.

### 2.2. Participant selection

Study participants were recruited through an online questionnaire distributed to patients diagnosed with diabetes. Social media and personal connections were used to encourage more participation in the survey. Eligible participants comprised those who met the criteria of being diabetic patients. Medical personnel were excluded from participation due to their advanced knowledge of the topic and to minimize response bias.

### 2.3. Sample size

The International Diabetes Federation reported that 18.7% of adults in Saudi Arabia had diabetes in 2021.^[17]^ Using this prevalence and the standard sample size formula (n = *Z*^2^ * *P* (1−*P*)/*d*^2^), the minimum sample size was calculated to be 232. To account for potential non-responses common in survey studies, an additional 10% of participants were included, resulting in 254 individuals who participated in the study.

### 2.4. Data collection procedure

The questionnaire was distributed electronically along with an introductory letter. The cover page of the questionnaire included a consent form, and participants were required to provide their informed consent before proceeding to complete the survey. Participation was voluntary, and respondents could only begin filling out the questionnaire after declaring their agreement to participate.

### 2.5. Questionnaire and its content

The questionnaire was developed in line with the objective of the study. The questionnaire comprised 2 main sections. The first section gathered socio-demographic information, including age, gender, education level, marital status, and body weight. The second section contained questions related to medication adherence, focusing on reasons for non-adherence, perceived health status, frequency and consistency, and medication-taking behavior. Out of 6 questions related to medication adherence, 5 required respondents to answer with “yes” or “no,” while the last statement allows responses such as “never,” “once a month,” “a few times a month,” “once a week,” or “more than once a week.”

The responses were scored to determine medication adherence. From question number 6 to question number ten, one point was assigned for each “no” response, and zero points were given for each “yes” response. For question number 11, however, the response with the statement “never” received one point, while the remaining options received zero. The overall score was 6. “Low adherent” was defined as having a score of less than 3, “intermediate adherent” as having a score between 3 and 4, and “high adherent” as having a score greater than 4.

Two research experts – one an associate professor from the University of Ha’il in Saudi Arabia and the other as a professor from the American University of Taxila in Guyana – validated the questionnaire’s face and content. After taking into account their insightful recommendations, the questionnaire was pretested among 20 participants who were later removed from the study. Some of the questions were simplified when their phrasing was discovered to be unclear during the piloting of the questionnaire. A question that was modified had a double-barrel meaning. Following the removal of one of the 2 items with comparable meanings, the Cronbach alpha score was 0.827, indicating good reliability (≥0.8) of the questionnaire.

### 2.6. Data handling and statistical analysis

Data collected from the questionnaires were extracted in the form of a Microsoft Excel spreadsheet and subsequently imported into IBM SPSS version 21.0 for statistical analysis. The Kolmogorov-Smirnov test was used to analyze the data for normality distribution. The *P*-value was <.001 which is <.05. This suggests that the data were non-normally distributed. The Mann–Whitney *U* test and Kruskal–Wallis test were used to see the association between the demographics of the participants and medication adherence scores; associations were considered significant at a *P*-value of ≤0.05.

### 2.7. Informed consent and ethical consideration

Every participant was made aware of the study’s objective. Prior to beginning data collection, each respondent gave their informed consent, and participation was entirely voluntary. Ethical approval was obtained from the Research Ethics Committee at the University of Ha’il, Saudi Arabia dated 22/5/2023 with approval number: H-2023-282.

## 3. Results

Demographic analysis revealed that about two-thirds (66.9%) of the participants represented age groups of 16 to 35 years and 56 to 75 years, and the majority (61.4%) of the participants were female. Although 23.6% of the participants had no formal education, a larger fraction (54.7%) attained either higher secondary or bachelor’s level of education. In terms of marital status, 55.5% were married (Table 1).

**Table 1:** Demography of participants (n = 254).

Variables	Frequency	Percentage
Age (yr)		
Under 16	17	6.7
16–35	90	35.4
36–55	64	25.2
56–75	80	31.5
76 & above	3	1.2
Gender		
Male	98	38.6
Female	156	61.4
Education		
No formal education	60	23.6
Primary level education	26	10.2
Secondary level education	19	7.5
Higher secondary	34	13.4
Bachelor	105	41.3
Post graduate	10	4
Marital status		
Unmarried	101	39.8
Married	141	55.5
Divorced	3	1.2
Widow	9	3.5

In general, patients faced problems frequently in terms of inconvenience in sticking to their treatment plan (77.2%), sometimes forgetting to take their diabetes medication as prescribed or directed (66.5%), and skipping taking their diabetes medicine due to other commitments such as being busy or away from home (54.7%). On the other hand, either 26.4% of the patients stopped their medication due to side effects of the medication or they started feeling better with their medication (Table 2). However, 61.4% of the patients sometimes faced difficulty remembering to take all of their diabetes medicines.

**Table 2:** Medication adherence among the participants (n = 254).

Statement	Yes n (%)	No n (%)
Do you occasionally miss to take your diabetes medication as prescribed?	169 (66.5)	85 (33.5)
Have you had any side effects from your diabetes medication that stop you from taking it?	67 (26.4)	187 (73.6)
Do you ever skip to take your diabetes medication due to other commitments, like being busy or away from home?	139 (54.7)	115 (45.3)
Have you ever stopped taking your diabetes medication when you start feeling better?	67 (26.4)	187 (73.6)
Have you ever missed your diabetes medication because it was inconvenient?	196 (77.2)	58 (22.8)

**Figure 1.**
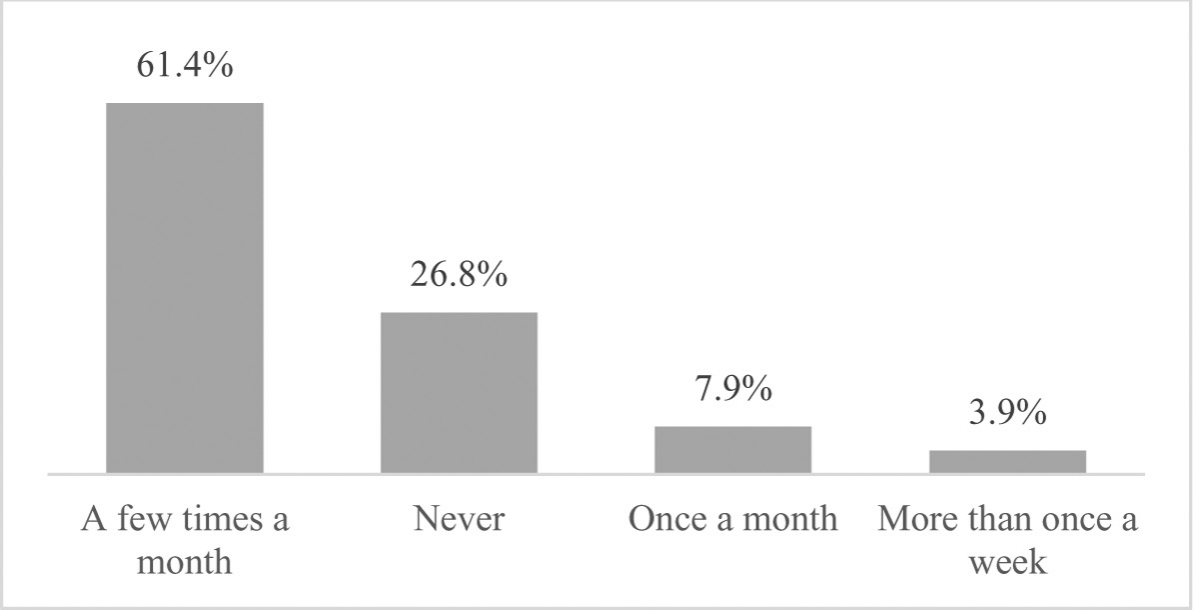
Difficulty in taking prescribed medications.

**Figure 2.**
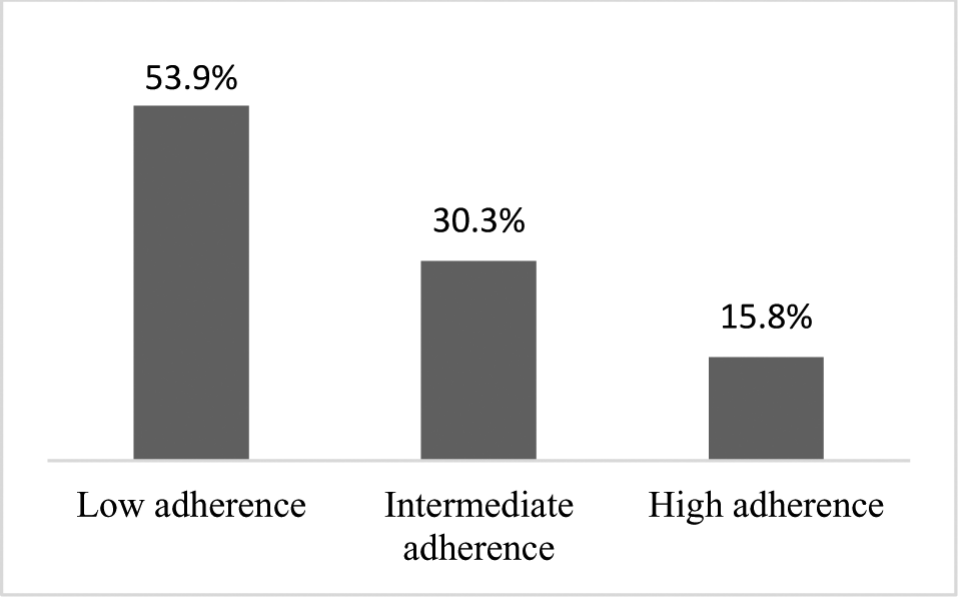
Medication adherence level.

Most participants (61.4%, n = 156) said they had trouble taking their prescription drugs a few times a month, while only 3.9% (n=10) said they had trouble more than once a week (Fig. 1). More than 84% (n = 214) of the patients were low-to-intermediate adherent to their antidiabetic medications, whereas 15.8% (n = 40) were highly adherent to their medications (Fig. 2).

Table 3 shows a significant association between age and medication adherence scores (*P*-value < .001). The highest percentage of participants with high adherence is among those aged 16 to 35 (47.5%), followed by those aged 36 to 55 (32.5%). Similarly, a significant association between education level and medication adherence scores was observed (*P*-value < .001). Participants with no formal education are more likely to have low adherence (38%), while those with secondary and higher education are more likely to have high adherence. On the other hand, no such associations were found between gender (*P* = .174) and body weight (*P* = .242) with medication adherence scores.

**Table 3:** Association between demographic characteristics of participants and medication adherence score (n = 254).

Variables	Medication adherence			
	Low adherence (137) n (%)	Intermediate adherence (77) n (%)	High adherence (40) n (%)	*P*-value
Age (yr)†				
Under 16	3 (2.2)	8 (10.4)	6 (15)	<.001*
16–35	34 (24.8)	37 (48.1)	19 (47.5)	
36–55	35 (25.5)	16 (20.8)	13 (32.5)	
56–75	62 (45.3)	16 (20.8)	2 (5)	
76 & above	3 (2.2)	0 (0)	0 (0)	
Gender‡				
Male	56 (40.9)	28 (36.4)	14 (35)	.174
Female	81 (59.1)	49 (63.6)	26 (65)	
Education†				
No formal education	52 (38)	8 (10.4)	0 (0)	<.001*
Primary level education	19 (13.9)	5 (6.5)	2 (5)	
Secondary level	4 (2.9)	6 (7.8)	9 (22.5)	
Higher secondary	13 (9.5)	13 (16.9)	8 (20)	
Bachelor	48 (35)	38 (49.4)	19 (47.5)	
Post graduate	1 (0.7)	7 (9.1)	2 (5)	
Marital status†				
Unmarried	37 (27)	39 (50.6)	25 (62.5)	.001*
Married	91 (66.4)	35 (45.5)	15 (37.5)	
Divorced	3 (2.2)	0 (0)	0 (0)	
Widow	6 (4.4)	3 (3.9)	0 (0)	
Weight†				
<40 kg (underweight)	0 (0)	2 (2.6)	0 (0)	.242
40–60 kg (normal weight)	26 (19)	26 (3.8)	7 (17.5)	
61–80 kg (over weight)	81 (59.1)	18 (23.4)	31 (77.5)	
81 kg & above (obese)	30 (21.9)	31 (40.3)	2 (5)	

* *P*-value ≤0.05 is considered statistically significant.

† Kruskal–Wallis test.

‡ Mann–Whitney *U* test.

## 4. Discussion

Diabetes mellitus is an emerging health problem in Saudi Arabia, and there is an urgent need to address this issue, including medication adherence. This study provides significant insights into medication adherence among diabetic patients in the Hail region.

The predominance of diabetes among mid-aged adults and the higher proportion of female participants in this study reflect commonly observed trends in Middle Eastern populations, and they influence health-seeking behaviors, including medication adherence.^[3,18,19]^ Inconvenience in following the treatment plan and occasional forgetfulness in taking diabetes medication were frequent challenges faced by the diabetes patients in this study. These findings align with global research that identifies forgetfulness and treatment inconvenience as the main barriers to medication adherence.^[20,21]^ Another issue that was noted was missing medication due to other obligations, which emphasizes how daily routines and lifestyle factors affect medication-taking behaviors.^[22,23]^ Adverse effects and the perception of recovery resulted in poor medication adherence. Similar perceptions have been documented in other studies, where adverse effects and perceived recovery lead to non-adherence.^[20–22,24,25]^ This is because patients may stop or avoid treatment due to discomfort or fear of negative side effects. Additionally, patients may become less inclined to adhere to the recommended regimen if they believe they have recovered or are no longer suffering symptoms. These results emphasize the significance of educating patients about the need for ongoing medication, even in cases where symptoms improve.

Younger individuals (16–35 years) were found to be more adherent, potentially owing to increased health awareness among the youth and a willingness to live longer and do something in their lives.^[26]^ Medication adherence was positively connected with higher educational attainment, most likely due to improved health literacy and an understanding of the significance of adhering to the dosage schedule. Numerous studies have found and advocated that health literacy has a positive relationship with medication adherence.^[27–29]^

Many studies show that family support promotes medication adherence by reminding spouses to take their medications and persuading them of the value of doing so in order to prevent long-term complications.^[22,30]^ On the other hand, this study found that single people took their diabetes drugs more consistently. Although it is unusual, this is possibly due to the fact that unmarried people may have fewer family responsibilities or caregiving duties.

Patients who were overweight or obese demonstrated greater medication adherence, despite a negligible correlation between body weight and medication adherence. This is in line with the findings of a study by Taher et al.^[31]^ This is due to their increased awareness of the health hazards linked with their high body weight and their increased caution regarding it.

### 4.1. Limitations and recommendations

The cross-sectional nature of this study prevents the establishment of causal relationships, and its dependence on self-reported data raises the possibility of bias. Furthermore, the use of an online survey and nonrandom sampling may have restricted participation to people with internet access, which could impact the findings’ representativeness and generalizability.

These results highlight the complexity of medication adherence, which is impacted by social, behavioral, and demographic factors. In order to increase adherence, particularly among younger and less educated groups, future research should focus on investigating the underlying causes of perceived discomfort and creating culturally appropriate interventions.

## 5. Conclusion

This research highlights the complex factors that affect medication adherence among diabetic patients in the Hail region of Saudi Arabia. The findings show that factors including body weight, age, and level of education are important barriers to medication adherence. Similarly, treatment inconvenience and forgetfulness are common obstacles that emphasize the need for customized approaches to improve compliance. It is essential to make an effort to correct misconceptions regarding side effects and to emphasize the significance of continued medication use. Enhancing patient education, streamlining medication regimens, and accounting for individual demographic and lifestyle characteristics can all lead to greater adherence and better health outcomes for diabetics in this area.

## Acknowledgments

The author wishes to express gratitude to the students for their assistance in collecting the data. Additionally, heartfelt appreciation is extended to the respondents; without their support, this study would not have been possible.

## Author contributions

**Conceptualization:** Mukhtar Ansari.

**Data curation:** Mukhtar Ansari.

**Formal analysis:** Mukhtar Ansari.

**Investigation:** Mukhtar Ansari.

**Methodology:** Mukhtar Ansari.

**Project administration:** Mukhtar Ansari.

**Resources:** Mukhtar Ansari.

**Software:** Mukhtar Ansari.

**Supervision:** Mukhtar Ansari.

**Validation:** Mukhtar Ansari.

**Visualization:** Mukhtar Ansari.

**Writing – original draft:** Mukhtar Ansari.

**Writing – review & editing:** Mukhtar Ansari.
